# Silencing circOMA1 Inhibits Osteosarcoma Progression by Sponging miR-1294 to Regulate c-Myc Expression

**DOI:** 10.3389/fonc.2022.889583

**Published:** 2022-04-13

**Authors:** Yubo Shi, Yunyun Tian, Xiangran Sun, Yonglong Qiu, Yingchun Zhao

**Affiliations:** Department of Orthopedics, Renmin Hospital of Wuhan University, Wuhan, China

**Keywords:** circOMA1, miR-1294, c-Myc, osteosarcoma, progression

## Abstract

**Background:**

Several studies have reported that circRNAs have a crucial function in the tumorigenesis of various cancers. However, the expression and function of circOMA1 in osteosarcoma is unknown.

**Methods:**

circOMA1 was identified through bioinformatics analysis. qRT-PCR was used to assess the expressions of circOMA1, miR-1294, and c-Myc in osteosarcoma tissues. Further, we performed functional experiments to explore the biological function of circOMA1 in osteosarcoma. Moreover, a luciferase reporter assay, RNA immunoprecipitation (RIP), and fluorescence *in situ* hybridisation (FISH) assay were performed to demonstrate the association between circOMA1 and miR-1294.

**Results:**

circOMA1 exhibited considerable upregulation in osteosarcoma tissues compared with adjacent normal tissues. Silencing circOMA1 suppressed osteosarcoma progression *in vitro* and *in vivo*. Mechanically, circOMA1 functioned as a sponge of miR-1294 to upregulate c-Myc expression.

**Conclusion:**

circOMA1 played the role of an oncogene in osteosarcoma and promoted osteosarcoma progression by mediating the miR-1294/c-Myc pathway, which might be a new target for treating osteosarcoma.

## Introduction

Osteosarcoma (OS) is a primary malignant bone tumour with an annual incidence of approximately 4 per million (1). OS is characterised by a highly aggressive and metastatic ability and is common in children and adolescents (2). With the rapid development of surgical techniques, neoadjuvant chemotherapy regimens, and immunotherapy techniques, the prognosis of patients with OS has been significantly improved (3–5). However, patients with OS experience chemotherapy resistance, recurrence, and severe adverse immune reactions, affecting their quality of life (6). Therefore, it is essential to study the molecular mechanism of OS pathogenesis and identify new targets to improve early diagnosis and targeted therapy.

Circular RNAs (circRNAs) have a closed-loop structure connected by covalent bonds and are highly abundant, stable, and conserved (7–9). CircRNAs can regulate gene transcription and splicing, encode or interact with proteins, and play the role of competitive endogenous RNAs (ceRNAs) to sponge miRNAs (10). According to some studies, circRNAs exert an indispensable function in tumorigenesis through sponging miRNAs. For example, circ-0074027 could promote the malignant phenotype of lung cancer through sponging miR-2467-3P to induce RhoA expression (11). In addition, Liu (12) reported 252 differentially expressed circRNAs between normal osteoblasts and osteosarcoma cell lines, which suggested that circRNAs are a new strategy and direction to explore the molecular targets of OS. Under the GSE96964 dataset, we observed that circOMA1 (hsa_circ_0002316) was strongly expressed in OS tissues compared with adjacent normal tissues. To the best of our knowledge, no study has reported the function of circOMA1 in OS. Therefore, the specific function of circOMA1 in developing osteosarcoma needs to be further explored.

In our study, using bioinformatics and molecular biology techniques, we explored the biological functions of circOMA1/miR-1294/c-Myc axis in tumorigenesis and development of OS, aiming to provide new targets and strategies for clinically treating osteosarcoma.

## Materials and Methods

### Tissue Samples

Eighteen paired OS specimens, and adjacent normal tissues were collected and stored at −80°C until further application. This work acquired approval from the Ethics Committee of Renmin Hospital of Wuhan University.

### Cell Culture and Transfection

Human osteoblast (hFOB1.19) and OS cell lines were cultured in DMEM with 10% foetal bovine serum (Gibco) at 37°C with 5% CO_2_. Short hairpin RNA over circOMA1 (sh-circOMA1) and the negative control shRNA (sh-NC) was obtained from Servicebo (Wuhan, China). miR-1294 mimics and negative control miRNA mimics (miR-NC) were synthesised by GenePharma (Shanghai, China). Lipofectamine (Invitrogen) was used to transfect these RNAs in OS cells per the manufacturer’s instructions.

### Cell Proliferation and EdU Assay

The transfected cells were cultured for 24, 48, and 72 h. Further, 10 µL CCK-8 solution was added to the 96-well plate, and the optical density (OD) was measured at 450 nm using a microplate reader. Further, a 5-ethynyl-2′-deoxyuridine (EdU) assay was performed to evaluate cell proliferation using Click-iT EdU-488 Kit (Servicebio, Wuhan, China).

### Transwell Assay and Wound Healing Assay

In transwell assay, 100 µL matrix gel was added to the upper chamber, and 600 µL DMEM with 10% FBS was added to the lower chamber. Subsequently, the cell suspension was added to the upper chamber and incubated for 2 days. Further, the cells were fixed, stained, and photographed. In the wound healing assay, the transfected cells were seeded in a 6-well plate at a density of 5 × 10^5^ cells per well. When the confluency reached 90%, a pipette tip was used to scratch a wound line. Photographs were taken to record the healing of scratches at different times.

### RNase R Treatment

Total RNA was treated with 3 U/mg of RNase R for 20 min. Further, qRT-PCR was performed for detecting the mRNA levels of circOMA1 and OMA1 mRNA.

### qRT-PCR

TRIzol reagent (Invitrogen) was used to extract total RNA from tissues or cells. Nano-Drop 2000 spectrophotometer was used to measure the concentration of RNA. cDNA was generated using a cDNA Synthesis Kit (Takara, China). The circRNA miRNA and mRNA levels were quantified using qPCR with SYBR Green (Takara, China), with U6 as the internal control for miRNA and GAPDH for circRNA and mRNA. The primers are listed in **Supplementary Table 1**.

### Western Blot

We extracted total proteins from tissues or cells from the lysis solutions (Servicebio, China). Next, the proteins were separated by SDS-PAGE and transferred to a PVDF membrane. Further, the membrane was rinsed with TBST and incubated with primary antibodies, namely, anti-c-Myc (Servicebio, China) and anti-β-actin (Servicebio, China). Subsequently, the membrane was incubated with secondary antibodies, and images were taken.

### Luciferase Reporter Assay

The circOMA1 and c-Myc 3′‐UTR sequences containing the wild‐type or mutant miR-1294 binding sites were amplified by PCR. These sequences were loaded to pmirGLO vectors to construct recombinant plasmids. Further, we co-transfected the recombinant plasmids and miR-1294 mimics or miR-NC to 143B and U2OS. Luciferase activity was measured using Luciferase Assay Kit (Beyotime, China).

### RNA Immunoprecipitation (RIP)

According to the standard protocol, the RIP assay was performed using Magna RIP™ RNA-Binding Protein Immunoprecipitation Kit (Milibo, USA). Further, qRT-PCR was performed to detect ircOMA1 and miR-1294 expressions in samples.

### Fluorescence *In Situ* Hybridisation (FISH) Assay

CircOMA1 probes and miR-1294 probes were synthesised by Servicebio (Wuhan, China). The cells were incubated with a hybridisation solution containing circOMA1 and miR-1294 probes overnight at 37°C. Confocal images were photographed using a Nikon Eclipse Ti microscope.

### Animal Experiments

Briefly, six BALB/c nude mice were classified into two groups, namely, sh-circOMA1 and sh-NC groups. In the sh-circOMA1 group, the 143B cells with a knockdown of circOMA1 were injected in mice, whereas 143B cells transinfected with negative control were subcutaneously injected in the sh-NC group. We measured and recorded the tumour volume every 5 days. After 30 days, the tumours were removed, weighed, and subjected to qRT-PCR.

### Bioinformatics Analysis

The circRNA expression profiles of OS were acquired from the GEO database. The interactions between circRNA and miRNA were predicted by Circinteractome and circBank, whereas miRNA–mRNA interactions were estimated using Targetscan, miRDB, and miRTarBase.

### Statistical Analysis

We used SPSS 13.0 software for data analysis. ANOVA was performed to evaluate the differences among more than two groups. The student’s unpaired t-test was used for unpaired comparisons, and paired t-test was used for paired comparisons. *P* < 0.05 was considered significant.

## Results

### circOMA1 Is Highly Expressed in OS Tissues and Cells

**Figure 1:**
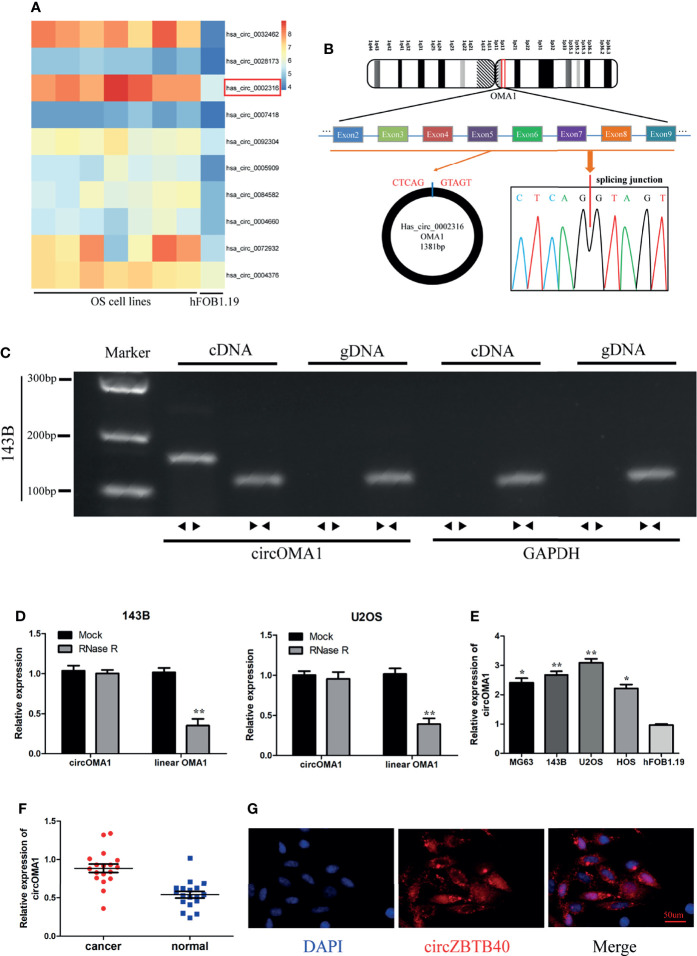
Characterisation and expression of circOMA1 in OS tissues and cell lines. **(A)** The heatmap exhibited DEcircRNAs in GSE96964. **(B)** The back-spliced region of circOMA1 was identified using Sanger sequencing. **(C)** Gel electrophoresis was performed to confirm the presence of circOMA1. **(D)** The expression of circOMA1 was assessed after RNase R treatment. **(E, F)** circOMA1 expression in OS tissues and cell lines. **(G)** We performed a FISH assay for detecting the location of circOMA1 in 143B cells. *P < 0.05, **P < 0.01.

Through analysing the GSE96964 dataset, it was observed that hsa_circ_0002316 (circOMA1) was highly denoted in OS cell lines (**Figure 1A**). circOMA1 is derived from 2–9 exons of the OMA1 gene on human chromosome 1. Subsequently, Sanger sequencing validated the circular structure of circOMA1 (**Figure 1B**). In addition, the circOMA1 was detected in cDNA with divergent primers but not in gDNA with divergent primers (**Figure 1C**). Similarly, the results revealed that circOMA1 had resistance to RNase R treatment, whereas linear OMA1 levels significantly decreased under RNase R treatment (**Figure 1D**). Furthermore, the expression of circOMA1 in OS tissues and cell lines (MG63, 143B, and U2OS) was assessed. circOMA1 expressions were notably denoted in OS cell lines and clinical samples (**Figures 1E, F**). Moreover, FISH assays demonstrated that circOMA1 was mainly localised in the cytoplasm (**Figure 1G**).

### Silencing circOMA1 Expression Inhibited OS Progression *In Vitro*

**Figure 2:**
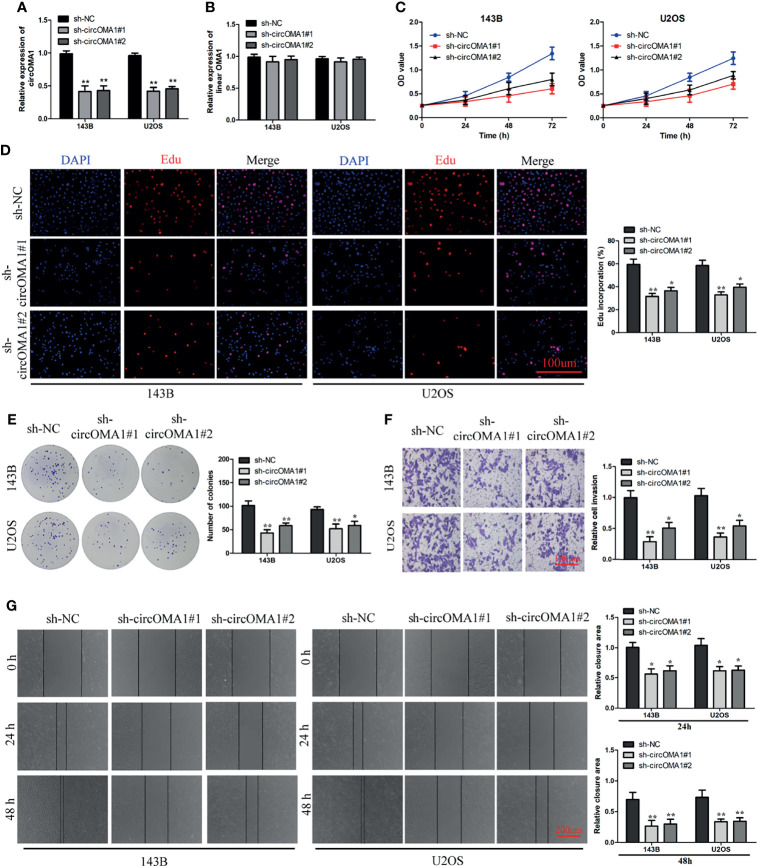
Knockdown of circOMA1 caused inhibition of OS progression. **(A, B)** The mRNA expression of circOMA1 and OMA1 in OS cells after transfection with sh-circOMA1 or sh-NC. **(C)** CCK8 assay was performed to assess cell viability. **(D, E)** Assessment of cell proliferation based on Edu **(D)** and colony formation assays **(E)** in OS cells. **(F)** The invasive ability of transfected cells was assessed using a transwell assay. **(G)** A wound-healing assay was performed to assess the migration of OS cells. *P < 0.05, **P < 0.01.

To investigate the role of circOMA1 in OS, two shRNAs (sh-circOMA1#1 and sh-cirCOMA1#2) were transfected into 143B and U2OS cells. circOMA1 expression was notably downregulated after transfection with sh-circOMA1 in 143B and U2OS cells (**Figure 2A**). However, the level of OMA1 mRNA remained unchanged (**Figure 2B**). CCK-8 assay results demonstrated that the cell viability of both cell lines in the sh-circOMA1 group was significantly inhibited compared with the sh-NC group (**Figure 2C**). Additionally, depletion of circOMA1 could inhibit the proliferation of 143B and U2OS cells (**Figures 2D, E**). Similarly, silencing circOMA1 could impair the migratory and invasive capacity of OS cells (**Figures 2F, G**). Therefore, these data revealed that silencing circOMA1 expression inhibited OS progression *in vitro*. Sh-circOMA1#1 was selected for subsequent study because it had a better silencing efficiency.

### circOMA1 Acts as a Sponge for miR-1294

**Figure 3:**
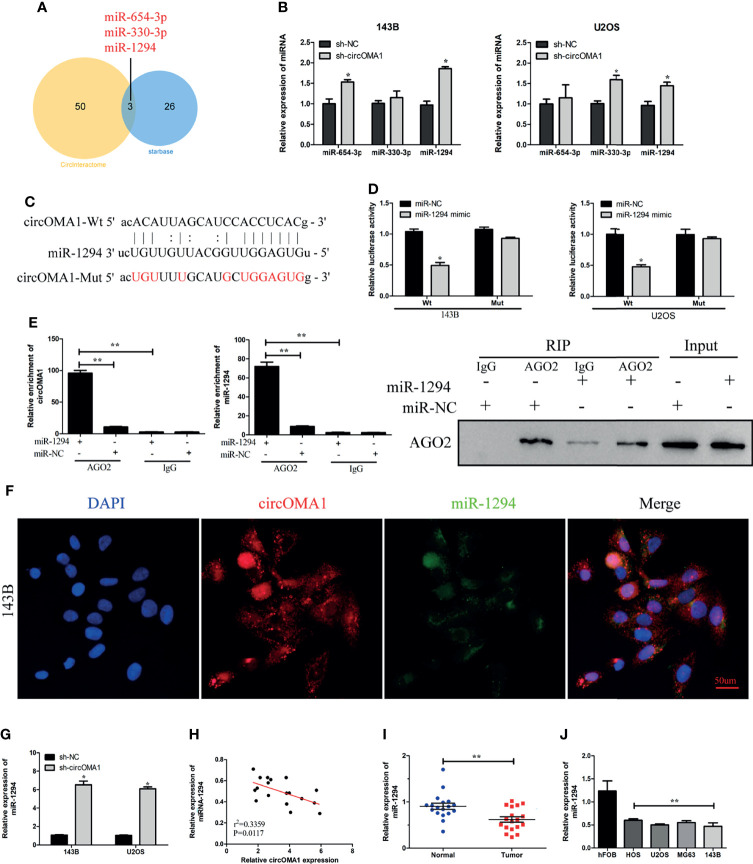
circOMA1 sponged miR-1294 in OS cells. **(A)** The Venn plot demonstrated the overlapping miRNAs screened by Starbase and CircInteractome. **(B)** The expression of three potential miRNAs in transfected cells. **(C)** The binding sites between circOMA1 and miR-1294. **(D)** Luciferase activity could be identified in OS cells after co-transfection. **(E)** The binding between circOMA1 and miR-1294 was verified by RIP assay and immunoblotting. **(F)** The relationship between circZBTB40 and miR-1270 was assessed by the FISH assay. **(G, H)** miR-1270 expression in OS tissues and cell lines. **(I)** MiR-1294 expression was examined in OS cells after the knockdown of circOMA1. **(J)** The relationship between circOMA1 and miR-1294. *P < 0.05, **P < 0.01.

The circRNA–miRNA interactions were predicted using bioinformatics analysis. Three miRNAs might bind to circOMA1 (**Figure 3A**). To investigate the interactions between circOMA1 and its potential targeted miRNAs, the expressions of miR-654-3p, miR-330-3p, and miR-1294 were evaluated in transfected cells (**Figure 3B**). Because miR-1294 was upregulated after the knockdown of circOMA1 in both cells, it was selected for further study. The data indicated that miR-1294 might bind to circOMA1 (**Figure 3C**). Moreover, miR-1294 mimicking lowered the luciferase activity in the wild-type construct but not in the mutant construct (**Figure 3D**). Similarly, the RIP assay revealed that circOMA1 and miR-1294 complex was enriched in the Ago2 group (**Figure 3E**). The FISH assay was used for detecting the co-localization between circOMA1 and miR-1294. The results demonstrated that circOMA1 and miR-1294 were co-localised in the cytoplasm (**Figure 3F**). Additionally, this study identified miR-1294 expression in OS tissues and cell lines. As expected, miRNA expression was decreased in both OS cell lines and tissues (**Figures 3G, H**). The qRT-PCR results revealed that knockdown of circOMA1 resulted in the upregulation of miR-1270 expression in OS cells (**Figure 3I**). Interestingly, miR-1294 expression negatively correlated with circOMA1 (**Figure 3J**). Collectively, these results demonstrated that circOMA1 played the role of a sponge for miR-1294.

### Downregulation of miR-1294 Reversed the Anti-Tumorigenic Impact of sh-circOMA1

**Figure 4:**
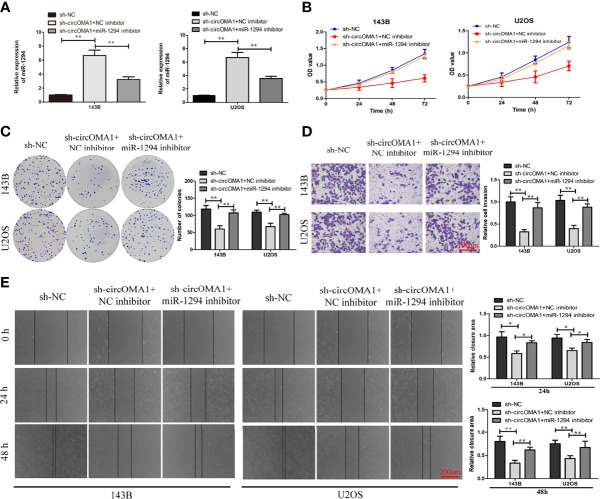
Downregulation of miR-1294 could reverse the anti-tumorigenic impact of sh-circOMA1. **(A)** The mRNA levels of miR-1294 were assessed using qRT-PCR. **(B, C)** CCK-8 and colony-forming assays evaluated the impact of miR-1294 on cell viability and proliferation. **(D, E)** The impact of miR-1294 on cell invasion and migration was detected by transwell and wound healing assays. *P < 0.05, **P < 0.01.

To investigate the interaction between miR-1294 and circOMA1, OS cells were transfected with sh-circOMA1 and miR-1294 inhibitors. The results showed that miR-1294 expression was notably upregulated in OS cells after transfection with sh-circOMA1, whereas the miR-1294 inhibitor reversed the effect (**Figure 4A**). CCK-8 and colony-forming assay results indicated that mi-1294 inhibitor weakened the impact of sh-circOMA1 on cell viability and proliferation (**Figures 4B, C**). Similarly, sh-circOMA1 remarkably inhibited the invasive and migrant abilities of OS cells, whereas the impact was reversed by a miR-1294 inhibitor (**Figures 4D, E**). Therefore, downregulation of miR-1294 reversed the anti-tumorigenic effect of sh-circOMA1.

### circOMA1 Regulates the c-Myc Expression *via* miR-1294

**Figure 5:**
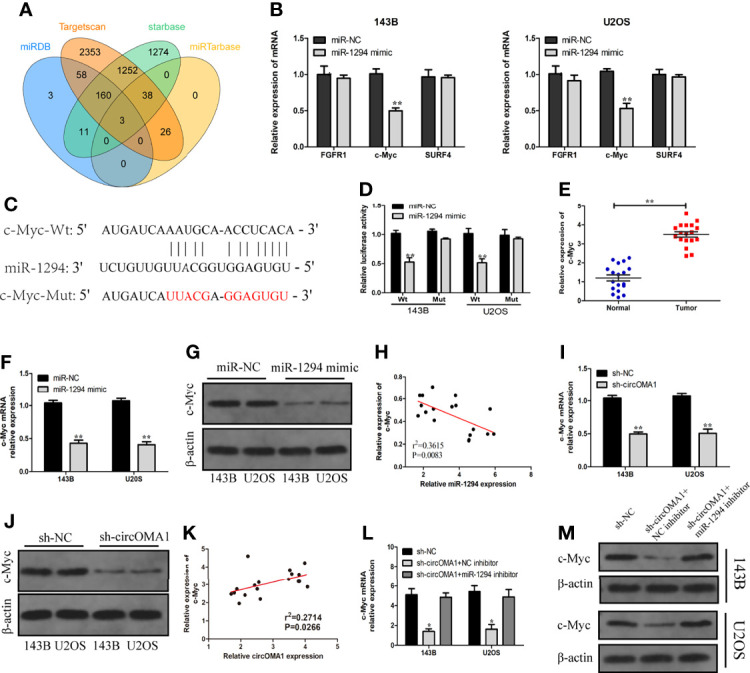
circOMA1 upregulated c-Myc expression *via* sponging miR-1294. **(A)** The intersection of possible target genes predicted by bioinformatics analysis. **(B)** The expressions of the potential target genes were assessed in OS cells after transfection with miR-1294 mimic or miR-NC. **(C)** The binding sites between miR-1294 and c-Myc. **(D)** Luciferase reporter assay confirmed the relationship between miR-1294 and c-Myc. **(E)** The expression of c-Myc in OS tissues. **(F, G)** MiR-1270 mimics decreased c-Myc mRNA and protein levels in OS cells. **(H)** The correlation between miR-1294 and c-Myc. **(I, J)** Sh-circOMA1 decreased c-Myc mRNA and protein levels in OS cells. **(K)** The relationship between circOMA1 and c-Myc. **(L, M)** The mRNA and protein levels of c-Myc in transfected cells. *P < 0.05, **P < 0.01.

Bioinformatics analysis revealed that FGFR1, c-Myc, and SURF4 were the three candidates for miR-1294 (**Figure 5A**). The expression of c-Myc was notably lowered in OS cells after transfection with miR-1294 mimic (**Figure 5B**). Targetscan database was used for detecting the binding sites between miR-1294 and circOMA1 (**Figure 5C**). Furthermore, the luciferase reporter assay confirmed the direct binding relationship between miR-1294 and c-Myc (**Figure 5D**). The c-Myc expressions in OS tissues were examined using qRT-PCR. It was observed that the expression of c-Myc was enhanced in OS tissues (**Figure 5E**). In addition, c-Myc mRNA and protein levels in both cells were downregulated following miR-1294 mimic (**Figures 5F, G**). Interestingly, c-Myc negatively correlated with miR-1294 expression (**Figure 5H**).

To explore whether circOMA1 could regulate c-Myc, the expression of c-Myc was identified after the knockdown of circOMA1. The obtained results indicated that silencing circOMA1 inhibited c-Myc mRNA and protein expressions in 143B and U2OS cells (**Figures 5I, J**). C-Myc exhibited a positive correlation with circOMA1 expression (**Figure 5K**). To further identify whether circOMA1 regulated c-Myc *via* miR-1294, sh-circOMA1 and miR-1294 inhibitor were co-transfected into OS cells. The reductions in the mRNA and protein levels of c-Myc after sh-circOMA1 transfection were reversed by transfection with miR-1294 inhibitor (**Figures 5L, M**). It can be concluded that circOMA1 controlled the c-Myc expression *via* miR-1294.

### Silencing circOMA1 Inhibited OS Growth *In Vivo*

**Figure 6:**
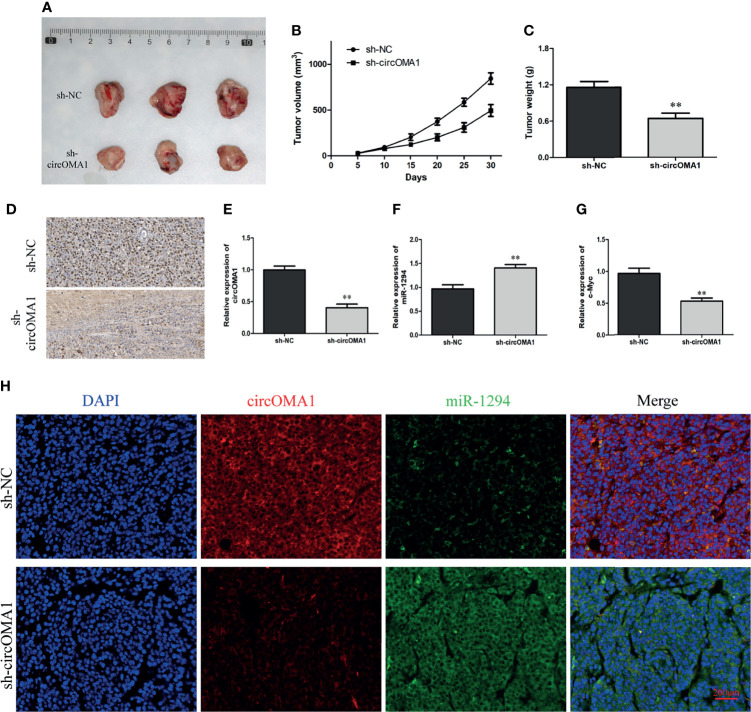
Silencing circOMA1 hindered the growth of tumour *in vivo*. **(A)** The images of tumour masses. **(B, C)** The tumour volume and weight are in two groups. **(D)** Ki-67 staining of tumour tissues. **(E–G)** The mRNA levels of circOMA1, miR-1294, and c-Myc were assessed using qRT-PCR. **(H)** FISH of circOMA1 and miR-1294 in tumors, **P < 0.01.

To further explore the impact of circOMA1 on OS cells *in vivo*, 143B cells transfected with sh-circOMA1 or sh-NC were injected subcutaneously in mice. The data indicated that silencing circOMA1 inhibited tumour volume and weight in the sh-circOMA1 group compared to the sh-NC group (**Figures 6A**–**C**). In addition, immunochemical findings revealed that the expression of the ki-67 protein was lowered in the sh-circOMA1 group (**Figure 6D**). According to qRT-PCR results, circOMA1 and c-Myc were downregulated in the sh-circOMA1 group, whereas miR-1294 expression exhibited upregulation in the sh-circOMA1 group (**Figures 6E**–**G**). The FISH assay showed that circOMA1 and miR-1294 were co-localised in the cytoplasm (**Figure 6H**).

## Discussion

CircRNA is a new type of non-coding RNA with various biological properties different from linear RNAs (13). Due to its unique circular structure, cirRNA has attracted increasing attention in recent years (14). Researchers have used second-generation sequencing and gene chip to screen for differentially expressed circRNAs in various tumours and observed that circRNAs have an essential function in tumour proliferation, metastasis, and drug resistance (15–18). For example, has_circ_0018414 accelerated malignant progression of lung adenocarcinoma *via* sponging miR-6807-3P (19). In addition, increasing evidence indicates that circRNA may be involved in tumorigenesis and progression as a tumour suppressor or activator (20–23).

OS is a normal aggressive malignant bone tumour (24). At present, the pathogenesis of OS is still unclear and believed to be complex and multifactorial (25). In addition, current treatment strategies are minimal for patients with early metastasis and recurrence, particularly in children and adolescents (26). Therefore, investigation of the potential mechanism of OS progression at the molecular level is urgently needed. circOMA1 is derived from 2–9 exons of the OMA1 gene on human chromosome 1.

Nevertheless, the biological role of circOMA1 in developing OS is not known. Through the analysis of the GSE96964 dataset, this study discovered that circOMA1 expression was notably upregulated in OS tissues compared with adjacent healthy tissues. It was further verified that circOMA1 was highly denoted in clinical samples of OS and cell lines. In addition, the results suggested that silencing circOMA1 impaired the migratory, invasive, and colony-forming abilities of OS cells. Moreover, animal experiments demonstrated that depletion of circOMA1 inhibited tumour growth. Collectively, these data proved that circOMA1 exhibited a carcinogenic function in developing OS.

Several studies confirmed that circRNAs regulate gene expression *via* sponging miRNA (27, 28). Therefore, we speculated that circOMA1 could modulate OS progression through this mechanism. Through bioinformatics analysis, we observed that miR-1294 might be a target of circOMA1. Further, FISH assay, a luciferase reporter assay, and RIP confirmed that circOMA1 could bind to miR-1294 in OS cells. Importantly, miR-1294 had a negative correlation with circOMA1 expression. These results demonstrated that circOMA1 played the role of a sponge for miR-1294.

C-Myc has been shown to have a vital function in tumorigenesis of various cancers (29, 30). Therefore, we investigated the relationship between cirOMA1, miR-1294, and c-Myc. Through luciferase reporter assay, we confirmed that c-Myc was a target of miR-1294. The finding was consistent with the results obtained by Rong et al., who verified the binding relationship between c-Myc and miR-1294 (31). Besides, the decrease in the mRNA and protein levels of c-Myc following circOMA1 shRNA transfection could be reversed by transfection with miR-1294 inhibitor. In addition, c-Myc expression exhibited a positive relationship with circOMA1, which verified the existence of a circOMA1/miR-1294/c-Myc axis in OS. These results indicated that circOMA1 could regulate the c-Myc expression *via* miR-1294.

In conclusion, this study suggested that circOMA1 played the role of an oncogene in OS and promoted OS progression by mediating the miR-1294/c-Myc pathway, which might be a novel target for OS therapy.

## Data Availability Statement

The original contributions presented in the study are included in the article/**Supplementary Material**. Further inquiries can be directed to the corresponding author.

## Ethics Statement

The studies involving human participants were reviewed and approved by Renmin Hospital of Wuhan University. The patients/participants provided their written informed consent to participate in this study.

## Author Contributions

YS and YT made contributions to the conception and manuscript drafting of this study. XS and YQ were responsible for data analysis. YZ revised the manuscript. All authors contributed to the article and approved the submitted version.

## Conflict of Interest

The authors declare that the research was conducted in the absence of any commercial or financial relationships that could be construed as a potential conflict of interest.

## Publisher’s Note

All claims expressed in this article are solely those of the authors and do not necessarily represent those of their affiliated organizations, or those of the publisher, the editors and the reviewers. Any product that may be evaluated in this article, or claim that may be made by its manufacturer, is not guaranteed or endorsed by the publisher.

## References

[1] Meltzer PS Helman LJ. New Horizons in the Treatment of Osteosarcoma. N Engl J Med (2021) 385(22):2066–76. doi: 10.1056/NEJMra2103423 34818481

[2] Yoshida A. Osteosarcoma: Old and New Challenges. Surg Pathol Clin (2021) 14(4):567–83. doi: 10.1016/j.path.2021.06.003 34742481

[3] Hecker-Nolting S Langer T Blattmann C Kager L Bielack SS. Current Insights Into the Management of Late Chemotherapy Toxicities in Pediatric Osteosarcoma Patients. Cancer Manag Res (2021) 13:8989–98. doi: 10.2147/cmar.S287908 PMC8647031 34880679

[4] Ottesen TD Shultz BN Munger AM Sibindi C Yurter A Varthi AG. Characteristics, Management, and Outcomes of Patients With Osteosarcoma: An Analysis of Outcomes From the National Cancer Database. J Am Acad Orthop Surg Glob Res Rev (2022) 6(2):e22.00009. doi: 10.5435/JAAOSGlobal-D-22-00009 PMC8865506 35192571

[5] Song XJ Bi MC Zhu QS Liu XL. The Emerging Role of lncRNAs in the Regulation of Osteosarcoma Stem Cells. Eur Rev Med Pharmacol Sci (2022) 26(3):966–74. doi: 10.26355/eurrev_202202_28006 35179763

[6] Meftahpour V Aghebati-Maleki A Fotouhi A Safarzadeh E Aghebati-Maleki L. Prognostic Significance and Therapeutic Potentials of Immune Checkpoints in Osteosarcoma. EXCLI J (2022) 21:250–68. doi: 10.17179/excli2021-4094 PMC8822307 35145371

[7] Huang W Wu Y Qiao M Xie Z Cen X Huang X. CircRNA-miRNA Networks in Regulating Bone Disease. J Cell Physiol (2022) 237(2):1225–44. doi: 10.1002/jcp.30625 34796958

[8] Yang G Wu Y Wan R Sang H Liu H Huang W. The Role of Non−Coding RNAs in the Regulation, Diagnosis, Prognosis and Treatment of Osteosarcoma (Review). Int J Oncol (2021) 59(3):69. doi: 10.3892/ijo.2021.5249 34296296

[9] Liu J Yang L Fu Q Liu S. Emerging Roles and Potential Biological Value of CircRNA in Osteosarcoma. Front Oncol (2020) 10:552236. doi: 10.3389/fonc.2020.552236 33251132 PMC7673402

[10] Li Z Li X Xu D Chen X Li S Zhang L. An Update on the Roles of Circular RNAs in Osteosarcoma. Cell Prolif (2021) 54(1):e12936. doi: 10.1111/cpr.12936 33103338 PMC7791175

[11] Duan Z Wei S Liu Y. Circ_0074027 Contributes to Non-Small Cell Lung Cancer Progression Through Positively Modulating RHOA *via* Sequestering miR-2467-3p. J Bioenerg Biomembr (2021) 53(2):223–33. doi: 10.1007/s10863-021-09876-6 33687619

[12] Liu W Zhang J Zou C Xie X Wang Y Wang B. Microarray Expression Profile and Functional Analysis of Circular RNAs in Osteosarcoma. Cell Physiol Biochem (2017) 43(3):969–85. doi: 10.1159/000481650 28957794

[13] Enuka Y Lauriola M Feldman ME Sas-Chen A Ulitsky I Yarden Y. Circular RNAs Are Long-Lived and Display Only Minimal Early Alterations in Response to a Growth Factor. Nucleic Acids Res (2016) 44(3):1370–83. doi: 10.1093/nar/gkv1367 PMC4756822 26657629

[14] Harrison DJ Geller DS Gill JD Lewis VO Gorlick R. Current and Future Therapeutic Approaches for Osteosarcoma. Expert Rev Anticancer Ther (2018) 18(1):39–50. doi: 10.1080/14737140.2018.1413939 29210294

[15] Li S Liu F Zheng K Wang W Qiu E Pei Y. CircDOCK1 Promotes the Tumorigenesis and Cisplatin Resistance of Osteogenic Sarcoma *via* the miR-339-3p/IGF1R Axis. Mol Cancer (2021) 20(1):161. doi: 10.1186/s12943-021-01453-0 34876132 PMC8650521

[16] Lou J Zhang H Xu J Ren T Huang Y Tang X. Circusp34 Accelerates Osteosarcoma Malignant Progression by Sponging miR-16-5p. Cancer Sci (2022) 113(1):120–31. doi: 10.1111/cas.15147 PMC8748222 34592064

[17] Mao X Guo S Gao L Li G. Circ-XPR1 Promotes Osteosarcoma Proliferation Through Regulating the miR-214-5p/DDX5 Axis. Hum Cell (2021) 34(1):122–31. doi: 10.1007/s13577-020-00412-z 32920730

[18] Qi X Zhang DH Wu N Xiao JH Wang X Ma W. ceRNA in Cancer: Possible Functions and Clinical Implications. J Med Genet (2015) 52(10):710–8. doi: 10.1136/jmedgenet-2015-103334 26358722

[19] Yao Y Zhou Y Hua Q. circRNA Hsa_Circ_0018414 Inhibits the Progression of LUAD by Sponging miR-6807-3p and Upregulating DKK1. Mol Ther Nucleic Acids (2021) 23:783–96. doi: 10.1016/j.omtn.2020.12.031 PMC7868730 33614229

[20] Qin G Wu X. Circular RNA Hsa_Circ_0032463 Acts as the Tumor Promoter in Osteosarcoma by Regulating the MicroRNA 498/LEF1 Axis. Mol Cell Biol (2021) 41(8):e0010021. doi: 10.1128/mcb.00100-21 34096776 PMC8300801

[21] Wang L Li B Yi X Xiao X Zheng Q Ma L. Circ_SMAD4 Promotes Gastric Carcinogenesis by Activating Wnt/β-Catenin Pathway. Cell Prolif (2021) 54(3):e12981. doi: 10.1111/cpr.12981 33458917 PMC7941240

[22] Wang Y Wo Y Lu T Sun X Liu A Dong Y. Circ-AASDH Functions as the Progression of Early Stage Lung Adenocarcinoma by Targeting miR-140-3p to Activate E2F7 Expression. Transl Lung Cancer Res (2021) 10(1):57–70. doi: 10.21037/tlcr-20-1062 33569293 PMC7867743

[23] Wu Y Xie Z Chen J Chen J Ni W Ma Y. Circular RNA Circtada2a Promotes Osteosarcoma Progression and Metastasis by Sponging miR-203a-3p and Regulating CREB3 Expression. Mol Cancer (2019) 18(1):73. doi: 10.1186/s12943-019-1007-1 30940151 PMC6444890

[24] Anderson ME. Update on Survival in Osteosarcoma. Orthop Clin North Am (2016) 47(1):283–92. doi: 10.1016/j.ocl.2015.08.022 26614941

[25] Zhao X Wu Q Gong X Liu J Ma Y. Osteosarcoma: A Review of Current and Future Therapeutic Approaches. BioMed Eng Online (2021) 20(1):24. doi: 10.1186/s12938-021-00860-0 33653371 PMC7923306

[26] Otoukesh B Boddouhi B Moghtadaei M Kaghazian P Kaghazian M. Novel Molecular Insights and New Therapeutic Strategies in Osteosarcoma. Cancer Cell Int (2018) 18:158. doi: 10.1186/s12935-018-0654-4 30349420 PMC6192346

[27] Wang J Zhao X Wang Y Ren F Sun D Yan Y. circRNA-002178 Act as a ceRNA to Promote PDL1/PD1 Expression in Lung Adenocarcinoma. Cell Death Dis (2020) 11(1):32. doi: 10.1038/s41419-020-2230-9 31949130 PMC6965119

[28] Zhang C Zhou H Yuan K Xie R Chen C. Overexpression of Hsa_Circ_0136666 Predicts Poor Prognosis and Initiates Osteosarcoma Tumorigenesis Through miR-593-3p/ZEB2 Pathway. Aging (Albany NY) (2020) 12(11):10488–96. doi: 10.18632/aging.103273 PMC7346030 32424109

[29] van Schaijik B Davis PF Wickremesekera AC Tan ST Itinteang T. Subcellular Localisation of the Stem Cell Markers OCT4, SOX2, NANOG, KLF4 and C-MYC in Cancer: A Review. J Clin Pathol (2018) 71(1):88–91. doi: 10.1136/jclinpath-2017-204815 29180509

[30] Peng Y Liu J Wang Z Cui C Zhang T Zhang S. Prostate-Specific Oncogene OTUD6A Promotes Prostatic Tumorigenesis via deubiquitinating and stabilizing c-Myc. Cell Death Differ (2022). doi: 10.1038/s41418-022-00960-x PMC9433443 35217790

[31] Rong Z Shi S Tan Z Xu J Meng Q Hua J. Circular RNA CircEYA3 Induces Energy Production to Promote Pancreatic Ductal Adenocarcinoma Progression Through the miR-1294/C-Myc Axis. Mol Cancer (2021) 20(1):106. doi: 10.1186/s12943-021-01400-z 34419070 PMC8379744

