# Supplementary material for: Silencing circOMA1 Inhibits Osteosarcoma Progression by Sponging miR-1294 to Regulate c-Myc Expression
Source: Front Oncol. 2022 Apr 13;12:889583. doi: 10.3389/fonc.2022.889583 (PMC9043560; doi:10.3389/fonc.2022.889583)
Supplement: Supplementary file 1 [file Table_1.docx]

| CircOMA1 | Forward | 5’- CAAATGGAGTTCGTTGATAGCCT -3’ |
| --- | --- | --- |
|  | Reverse | 5’- CGTGAGGTGGATGCTAATGTGT -3’ |
| miR-1294 | Forward | 5’- GCGTGTGAGGTTGGCATTG -3’ |
|  | Reverse | 5’- AGTGCAGGGTCCGAGGTATT -3’ |
| c-Myc | Forward | 5’- GTGCTCCATGAGGAGACACCG -3’ |
|  | Reverse | 5’- CAGACTCTGACCTTTTGCCAGG -3’ |
| GAPDH | Forward | 5’- CCTGCCGGTGACTAACCCTG -3’ |
|  | Reverse | 5’- TCCACCACTGACACGTTGGC -3’ |

Supplementary table 1. Primer sequences in this study
